# Cost-effectiveness analysis of ibrutinib in patients with Waldenström macroglobulinemia in Italy

**DOI:** 10.1080/20016689.2017.1393308

**Published:** 2017-11-07

**Authors:** Andrea Aiello, Anna D’Ausilio, Roberta Lo Muto, Francesca Randon, Luca Laurenti

**Affiliations:** ^a^ Price and Market Access Department, Creativ – Ceutical, Milan, Italy; ^b^ Health Economics Market Access Reimbursement Department, Janssen-Cilag, Cologno Monzese, Italy; ^c^ Department of Hematology, Catholic University of Rome, “A. Gemelli” Hospital, Rome, Italy

**Keywords:** Waldenström Macroglobulinemia, cost-effectiveness analysis, ibrutinib, Italy

## Abstract

**Background and Objective:** Ibrutinib has recently been approved in Europe for Waldenström Macroglobulinemia (WM) in symptomatic patients who have received at least one prior therapy, or in first-line treatment for patients unsuitable for chemo-immunotherapy. The aim of the study is to estimate the incremental cost-effectiveness ratio (ICER) of ibrutinib in relapse/refractory WM, compared with the Italian current therapeutic pathways (CTP).

**Methods:** A Markov model was adapted for Italy considering the National Health System perspective. Input data from literature as well as global trials were used. The percentage use of therapies, and healthcare resources consumption were estimated according to expert panel advice. Drugs ex-factory prices and national tariffs were used for estimating costs. The model had a 15-year time horizon, with a 3.0% discount rate for both clinical and economic data. Deterministic and probabilistic sensitivity analyses were performed to test the results strength.

**Results:** Ibrutinib resulted in increased Life Years Gained (LYGs) and increased costs compared to CTP, with an ICER of €52,698/LYG. Sensitivity analyses confirmed the results of the BaseCase. Specifically, in the probabilistic analysis, at a willingness to pay threshold of €60,000/LYG ibrutinib was cost-effective in 84% of simulations.

**Conclusions:** Ibrutinib has demonstrated a positive cost-effectiveness profile in Italy.

## Introduction

Waldenström Macroglobulinemia (WM) is a B-cell lymphoproliferative disorder characterized by high levels of immunoglobulin M (IgM [macroglobulin]) in peripheral blood and histological bone marrow with evidence of at least 10% lymphoplasmacytic, associated or not with lymphadenopathy and/or splenomegaly [1–6]. High levels of IgM may be responsible for hyperviscosity syndrome but are generally associated with typical fundoscopic findings [2]. It is a rare disease of the elderly, with a median patients’ age of 65 years and a slight predominance of males over females [2,4,7]. The annual incidence in a European standard population was estimated to be 7.3 and 4.2 per million in males and females, respectively [8]. Although indolent, WM remains incurable and despite the probability of survival for several years, even decades, patients suffer from multiple relapses that adversely affect their quality of life (QoL) and activities of daily living [1,9,10].

The treatment of WM is not standardized, and the choice of therapy is highly personalized, determined by the age, symptoms, comorbidities or preferences of the patient [9,11].

Unlike traditional cytotoxic chemotherapy, which affects both tumor cells and healthy cells, the targeted therapy agent ibrutinib focuses on tumor cells and prevents the kinases from being able to signal this tumor cell growth and division [12]. Ibrutinib is intended for selected hematologic cancers; it is a first-in-class, potent, orally administered drug, that covalently binds to Bruton’s tyrosine kinase (BTK) and inhibits B-cell antigen receptor signalling downstream of BTK [13]. Ibrutinib acts by blocking B-cell antigen receptor signalling, thereby reducing malignant proliferation of B-cells and inducing cell death [13].

Ibrutinib was approved for the first time in 2013 by the US Food and Drug Administration (FDA) and in 2014 by the European Medicine Agency (EMA), with the special status of orphan drug. Ibrutinib was previously indicated, as single agent, for the treatment of adult patients with: relapsed or refractory mantle cell lymphoma (MCL), previously untreated chronic lymphocytic leukaemia (CLL). It was also indicated as single agent or in combination with bendamustine + rituximab in patients with CLL who have received at least one prior therapy [14,15].

In 2015 ibrutinib was approved by FDA and EMA for its use in adults who have received previous treatment for their disease, or in previously untreated patients for whom treatment with chemo-immunotherapy is not suitable [9].

The main study in WM showed that monotherapy with ibrutinib was highly active, associated with durable responses, and safe in pre-treated patients with an overall response rate (ORR) of 90.5%, a two-year progression-free survival (PFS) and overall survival (OS) rates of 69.1% and 95.2%, respectively [16].

The emergence of ibrutinib, and other similar drugs, expands treatment options especially in those diseases that are still missing a standard of care; however, the cost of these is a major concern for healthcare payers [17].

The aim of this study is to estimate, from the Italian National Health System (NHS) perspective, the incremental cost-effectiveness ratio (ICER) of ibrutinib in relapsing/refractory (R/R) WM, compared with the current therapeutic pathways (CTP) applied to WM: fludarabine + cyclophosphamide + rituximab (FCR), bortezomib + rituximab (BOR), rituximab + cyclophosphamide + doxorubicin + vincristine + prednisone (RCHOP), bortezomib + dexamethasone + rituximab (BDR), dexamethasone + rituximab + cyclophosphamide (DRC), and bendamustine/rituximab (BR) [1,18–23].

## Material and methods

**Figure 1.**
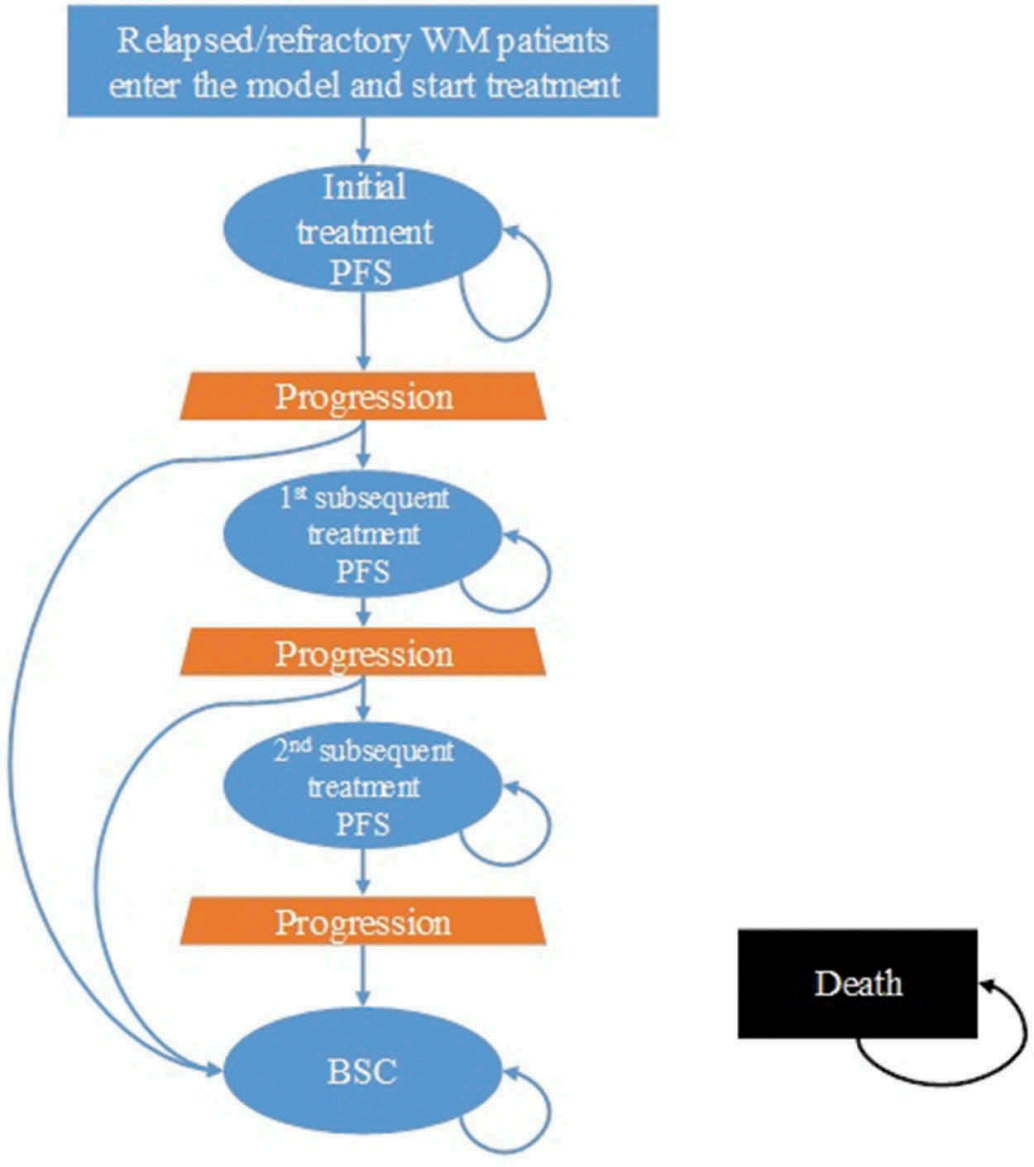
Markov model: general model structure. BSC = Best Supportive Care; PFS = Progression Free Survival; WM = Waldenström Macroglobulinemia.

A Markov model (Figure 1), previously developed to estimate the costs and outcomes associated with WM treatments, was adapted to the Italian healthcare setting.

The model follows patients in a 15-year time-horizon through five health states: initial treatment, first subsequent treatment, second subsequent treatment, best supportive care (BSC),^1^ ^1^Non-active form of treatment for symptoms management. and death. Transition probabilities were used to distribute patients to each health state. Patients enter the model as R/R WM patients in the initial treatment state, and transition out of the health state once progression triggers a treatment change or death occurs. Patients experiencing disease progression will receive subsequent treatment and experience a subsequent progression-free phase, while those refractory to treatment will receive BSC. Patients may be lost due to death during any of the phases.

Costs and health effects are assigned to each health state in a four-week model cycle. As the model progresses cycle-by-cycle for the duration of the time-horizon, costs and utility data are summed per treatment arm, allowing for the calculation of incremental costs and effectiveness per comparator at model completion.

A comparison of patient level data from the 1118e trial and a chart audit (on 452 samples recruited across nine European countries, including Italy, with up to five lines of treatment) were conducted to estimate the efficacy of ibrutinib versus current practice [16]. A cohort of 175 patients was created such that patients had a mix of prior lines of therapy so as to be comparable. Each patient was randomly sampled, so that the mix of therapy lines matched the trial, while the same patient was not allowed to be in two lines.

Clinical input data (efficacy and safety), treatment dosing schedules, overall population mortality were used to populate the model and derived respectively from global trials (2007–2015) as well as published literature [1,16,18–24]. Comparative efficacy for ibrutinib vs. CTP was derived from a multivariate Cox proportional hazard model performed to estimate the hazard ratio (HR) of the PFS for ibrutinib vs. CTP [1,16,18–24]. Missing characteristics data were imputed to ensure sufficient sample size. Given that only three patients died in the ibrutinib trial, it was not feasible to estimate relative treatment effect of ibrutinib on OS. For the estimation of PFS, a Weibull parametric fitting distribution for long-term projection was used.

The percentage use of ibrutinib and other WM therapies, as well as healthcare resources consumption in Italy were estimated according to a panel of clinicians who are experts in the management of WM (Table 1). The Italian model assumed a patient with an average weight of 75 kg and a body surface area of 1.8 m^2^. Health care resources consumption (routine visits and laboratory/instrumental tests, management of adverse events) were costed with both national inpatient and outpatient hospital tariffs while for drugs, ex-factory prices (Euro – €), updated in October 2016, were used [25,26]. The Activity-Based Costing (ABC) methodology was used to estimate the mean yearly cost of the compared patient pathways (Tables 2–4) [27–29]. A 3.0% rate to discount both clinical and economic data was used, as indicated by Italian guidelines [30].

Model outcomes are expressed in terms of incremental costs per life year gained (LYG). We did not consider quality adjusted life years (QALYs), as the experts could not validate the utility values reported in the model.

**Figure 2.**
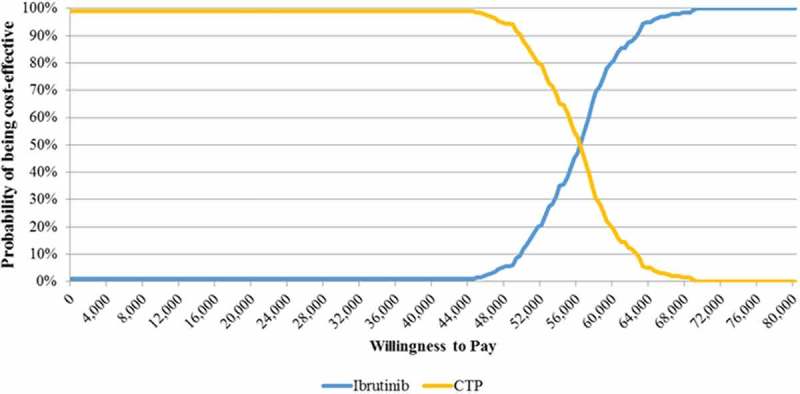
Probabilistic sensitivity analysis. CTP = Current Treatment Pathway.

To evaluate the robustness of the model and the results in the Base Case scenario (Table 5), both deterministic (DSA) and probabilistic sensitivity analyses (PSA) were performed (Table 6; Figure 2). In the DSA, parameters were changed through upper and lower bound values (Table 6). In the PSA (Figure 2), where all the variables are changed at the same time, a threshold of €60,000/LYG, in accordance with Italian publications [31], was used to estimate the willingness to pay (WTP) of a healthcare payer for ibrutinib in the treatment of WM.

**Table 1.** Patients (%) in the cohort analysis treated with different pathways in the CTP group.

Therapeutic Pathway	Pre Progression	Post Progression	Reference
FCR	10%	7%	Expert data
BOR	2%	2%	Expert data
RCHOP	17%	0%	Expert data
BDR	6%	5%	Expert data
DRC	24%	21%	Expert data
BR	41%	35%	Expert data
Total	100%	70%	

Legend: BDR = bortezomib + dexamethasone + rituximab; BOR = bortezomib + rituximab; BR = bendamustine/rituximab; CTP = Current Treatment Pathway; DRC = dexamethasone + rituximab + cyclophosphamide; FCR = fludarabine + cyclophosphamide + rituximab; RCHOP = rituximab + cyclophosphamide + doxorubicin + vincristine + prednisone.

**Table 2.** Drug unit cost.

Drug	Unit Dose	Cost	Reference
Ibrutinib	140 mg	67.40 €	[26]
Bendamustine	25 mg	46.41 €	[26]
Bortezomib	3.5 mg	1,300.00 €	[26]
Cyclophosphamide	500 mg	6.74 €	[26]
Dexamethasone	4 mg	0.86 €	[26]
Doxorubicin	50 mg	37.91 €	[26]
Fludarabine	25mg	76.74 €	[26]
Prednisone	5 mg	0.09 €	[26]
Rituximab	100 mg	277.60 €	[26]
Vincristine	1 mg	6.80 €	[26]

**Table 3.** Hospital inpatient unit costs for severe adverse events.

Severe Adverse Events	DRG Code	Tariff	Reference
Anaemia	395	1,676 €	[25]
Leucopoenia	399	1,704 €	[25]
Neutropenia	399	1,704 €	[25]
Thrombocytopenia	397	2,748 €	[25]
Lymphocytopenia	399	1,704 €	[25]
Non pulmonary infections	423	4,155 €	[25]
Neuropathy	19	1,210 €	[25]
Pulmonary toxicity	93	2,229 €	[25]
Constipation	183	959 €	[25]
Diarrhoea	183	959 €	[25]

Legend: DRG = Disease Related Group

**Table 4.** Other hospital and outpatient unit costs.

Other Variables	Tariff	Reference
Chemo therapy Administration*	371.00 €	[25]
Plasmapheresis	438.99 €	[25]
Full blood count	5.75 €	[25]
Immunoglobulin	12.42 €	[25]
Ultrasound	17.56 €	[25]
Chemistry	7.31 €	[25]
Albumin + Bilirubin	3.96 €	[25]
Phosphatase Alkaline	1.04 €	[25]
ALT + AST	2.04 €	[25]
Total Protein	1.13 €	[25]
Hematologic visit	20.66 €	[25]

*DRG code 410 – Day hospital tariff

**Table 5.** Base case analysis.

Variables	Ibrutinib (A)*	CTP (B)*	Δ (A-B)*
Life Years	6.77 LYGs	3.77 LYGs	+ 3.00 LYGs
Drug cost	200,461 €	33,835 €	+ 166,626 €
Administration cost	0 €	6,098 €	− 6,098 €
Serious Adverse Events costs	13,423 €	21,978 €	− 8,555 €
Total post-progression costs	19,182 €	12,957 €	+ 6,225 €
Total Costs	233,066 €	74,868 €	+ 158,198 €
ICER €/LY (ibrutinib vs CTP)*	52,698 €/LYG		

*Rounded numbers from the model simulation.

Legend: CTP = Current Treatment Pathway; ICER = Incremental Cost-Effectiveness Ratio; LYG = Life Year Gained

**Table 6.** Deterministic sensitivity analysis.

		Total Costs (€)*			Total LYG*			ICER € per LYG*	
Parameter	Alternative Inputs	Ibrutinib	CTP	Δ	Ibrutinib	CTP	Δ	Ibrutinib vs CTP	Δ from baseline
Base Case		233,066 €	74,868 €	+ 158,198 €	6.77 LYGs	3.77 LYGs	+ 3.00 LYGs	52,698 €/LYG	
Time Horizon	10 years	231,338 €	74,850 €	+ 156,489 €	6.13 LYGs	3.55 LYGs	+ 2.59 LYGs	60,497 €/LYG	+ 14.9%
	20 years	233,131 €	74,873 €	+ 158,257 €	6.96 LYGs	3.83 LYGs	+ 3.13 LYGs	50,631 €/LYG	− 3.9%
Health discount	0.0%	233,066 €	74,868 €	+ 158,198 €	7.80 LYGs	4.21 LYGs	+ 3.59 LYGs	44,015 €/LYG	−16.5%
Cost discount	0.0%	251,779 €	76,157 €	+ 175,622 €	6.77 LYGs	3.77 LYGs	+ 3.00 LYGs	58,503 €/LYG	+ 11.0%
PFS projection approach	Loglogistic	288,738 €	74,990 €	+ 213,748 €	7.37 LYGs	3.76 LYGs	+ 3.60 LYGs	59,294 €/LYG	+ 12.5%
Hazard Ratio for PFS	0.22	233,066 €	74,747 €	+ 158,318 €	6.77 LYGs	3.80 LYGs	+ 2.97 LYGs	53,385 €/LYG	+ 1.3%
	0.19	233,066 €	74,420 €	+ 158,645 €	6.77 LYGs	3.86 LYGs	+ 2.91 LYGs	54,526 €/LYG	+ 3.5%
Lines of subsequent treatment	0	214,540 €	62,363 €	+ 152,177 €	6.77 LYGs	3.77 LYGs	+ 3.00 LYGs	50,693 €/LYG	−3.8%
Post-progression efficacy	−20%	231,774 €	73,993 €	+ 157,780 €	6.34 LYGs	3.45 LYGs	+ 2.89 LYGs	54,507 €/LYG	+ 3.4%
	+ 20%	234,639 €	75,934 €	+ 158,705 €	7.32 LYGs	4.19 LYGs	+ 3.13 LYGs	50,709 €/LYG	−3.8%
Ibrutinib drug cost	−20%	192,973 €	74,868 €	+ 118,105 €	6.77 LYGs	3.77 LYGs	+ 3.00 LYGs	39,343 €/LYG	−25.3%
	+ 20%	273,158 €	74,868 €	+ 198,290 €	6.77 LYGs	3.77 LYGs	+ 3.00 LYGs	66,054 €/LYG	+ 25.3%

*Rounded numbers from the model simulation.

Legend: CTP = Current Treatment Pathway; LYG = Life Year Gained; PFS = Progression Free Survival.

## Results

### Base case

In the Cox regression, treatment with ibrutinib was the only covariate found to be statistically significant with a HR of 0.25 (95% confidence interval 0.11–0.57; *p* = 0.001). This is probably due to the relatively small number of progression events and the short follow-up in the trial. The HR for ibrutinib treatment is used in model base case analysis to inform the comparative efficacy of ibrutinib on PFS.

According to literature data [1,16,18–24], the model estimated an incremental value of + 3.0 LYGs vs. CTP (6.77 vs. 3.77) (Table 5). On the other hand, as expected, the group treated with ibrutinib showed higher healthcare costs, with an incremental total cost of +€158,198 (€233,066 vs. €74,868) compared to CTP.

The main cost driver, in both arms, was represented by drug cost, with an increment of +€166,626 with ibrutinib vs. CTP. However, such cost was partly offset by a reduction in other healthcare costs such as the management of serious AEs (Table 5).

In the base case, the deterministic cost-effectiveness analysis showed a favourable ICER, according to Italian WTP threshold [31] for ibrutinib of €52,698/LYG (Table 5).

### Deterministic sensitivity analysis

The DSA (Table 6) demonstrated the strength of the results in the base case. The variation in ibrutinib price (±20% vs. base case) was the main driver in the DSA, with ICERs between €39,343 and €66,054 per LYG. In the majority of the simulations, ICERs are close to the base case results. Particularly when lower HRs were used for the PFS, the ICERs had variations vs. the base case from +1.3% to +3.5%. Apart from the scenarios with a higher price of +20% for ibrutinib and a 10-year time-horizon, all the simulations showed ICERs below a WTP acceptability threshold of €60,000/LYG [31].

### Probabilistic sensitivity analysis

The probabilistic sensitivity analysis confirmed the strength of the results. At a WTP threshold of €60,000/LYG [31], ibrutinib was cost-effective in 81% of the simulations (Figure 2) and over a threshold of €68,800/LYG, which can be considered acceptable for a rare disease [32], in 100% of the cases.

## Discussion and conclusions

WM is treated most often with rituximab as a monotherapy or in combination with alkylating agents or nucleoside analogues. However, not one of these options is curative and standard of care has not been established [33]. Ibrutinib, a first in-class inhibitor of BTK, displays a unique targeted mechanism of action by inhibiting downstream signalling after the interaction between the mutated MYD88 (Leu265pro) protein, present in more than 90% of patients with WM, and BTK [33–35]. Agents such as rituximab (alone or in combination with bortezomib or bendamustine or fludarabine), do not target disease-specific abnormalities in WM, lack efficacy in WM, and can be associated with serious AEs, particularly in older adults [36]. Given that WM is associated with long survival and generally affects elderly people, aggressively intensifying therapy may not be useful in this population due to potentially life-threatening AEs [37]. The development of second primary malignancies (e.g. myelodysplastic syndrome, acute myeloid leukaemia) from prolonged chemotherapy treatment are, especially for fludarabine based regimens, of particular concern in patients with WM [38,39]. Moreover, the chronic utilization of non-specific developed pharmacological treatment could lead to serious AEs in WM patients with a huge economic impact on healthcare expenditure.

The pivotal, single-arm, phase II trial in previously treated patients with WM who were given ibrutinib had an overall response of 91%, defining ibrutinib as the most active single agent for relapsed or refractory WM to date [16,33].

Given that WM affects few patients, the economic consequences of WM have not been well characterized in the literature. Only a few economic studies were identified while neither cost-effectiveness analyses nor economic evaluations on ibrutinib for WM, published in full, were found [2,40].

Olszewski et al. in 2016 estimated that novel treatments, adopted as a standard of care, may lead to observable changes in both survival and costs of therapy [40]. In the study, mean total Medicare payments for the care of a patient with WM at 15 years from diagnosis were calculated to be $163,432 (€147,301).^2^ ^2^Conversion rate: €1 = $1.10951; Banca d’Italia. Exchange Rates Archives daily publication and historical series. 2015. Available at https://www.bancaditalia.it/compiti/operazioni-cambi/archivio-cambi/index.html?com.dotmarketing.htmlpage.language=1 Accessed November 2016. For the subgroup of patients who received chemotherapy, these costs were nearly twice as high at $193,150 (€174,086)^2^ compared with those not treated with chemotherapy at $106,705 (€96,173)^2^. This shift occurred immediately after year 2000 and coincided with widespread rituximab use [40].

A previous Italian study of Annibali et al. in 2005 evaluated only the costs of chemotherapy in patients with WM expressed as cost per unit of surface area for each treatment protocol [2]. In this study of 72 newly-diagnosed patients with WM in Italy, the cost per course of therapy varied from $16/m^2^ (€14/m^2^) for oral melphalan/cyclophosphamide/prednisone to $11,091/m^2^ (€9,996/m^2^)^2^ for cladribine/cyclophosphamide/rituximab. This study did not take into account the medical costs and costs of complications, but only chemotherapy costs [2].

In contrast to previous publications, the cost-effectiveness analysis presented in this paper aimed to describe the total costs and clinical benefits related to the introduction of ibrutinib in the treatment pathways and also the global costs and effects for patients treated with CTP in the Italian setting. Our analysis showed that despite the higher costs of ibrutinib pathway vs. CTP, the ICERs both in the Base Case and in DSA were under the threshold of €60,000/LY indicated as acceptable for Italy, except in the scenario with an increased price of +20% per ibrutinib and a 10-year time-horizon (Table 6). However, if we consider an equal value for LYGs and QALYs, due to a lack of evidence on quality of life data for Italian patients, with a WTP threshold for orphan drugs of £50,000/QALY (€68,885/QALY),^3^ ^3^Conversion rate: €1 = £0.72585; Banca d’Italia. Exchange Rates Archives daily publication and historical series. 2015. Available at https://www.bancaditalia.it/compiti/operazioni-cambi/archivio-cambi/index.html?com.dotmarketing.htmlpage.language=1 Accessed November 2016. as reported by Drummond et al. [32], the result is that in every scenario ibrutinib is cost-effective. Also the PSA confirmed the good pharmacoeconomic results of ibrutinib vs. CTP, with a probability of being cost-effective in 81% of the simulations at a WTP threshold of €60,000/LYG (Figure 2).

Due to the data limitations, the model analysis was subject to a few key uncertainties. The PFS of ibrutinib is projected based on immature Kaplan-Meier data as reported in the 1118e trial; therefore the long-term projection is subject to uncertainty, with a lack of definitive long-term clinical evidence. The mortality of ibrutinib during PFS was assumed to be the same as the general population mortality [24]. Given that only three deaths were reported in the 1118e trial [16], long-term projection of the trial data was not feasible. The mortality during PFS was used to determine the number of patients who would receive subsequent treatments, and consequently drove the post-progression survival. This assumption should be revised when longer follow-up becomes available for ibrutinib-treated patients. On the other hand, it is important to highlight that both the sensitivity analyses stressed the uncertainties of the model and that results are close to the Base Case.

The other relevant assumption is related to the time-horizon. WM is an indolent disease so, according to experts, the scenario with a 20-year time-horizon would be more realistic than the 15-year time-horizon. A longer time-horizon would produce more positive results for ibrutinib (lower ICER); however, we preferred to keep a conservative approach.

The last important issue regarding the cost-effectiveness analysis is the perspective used in the model. If the model had considered the societal perspective, and therefore the indirect costs, ibrutinib would have shown a more positive pharmacoeconomic profile. Ibrutinib has an oral method of administration to the intravenous CTP: patients treated with ibrutinib would need less travelling to the hospital and probably caregivers would incur lower productivity loss to accompany patients needing intravenous CTP or emergency visits due to CTP adverse events.

In conclusion, our study shows that with the use of ibrutinib in patients with WM, less effective palliative therapies and chemotherapy-related AEs can be avoided. In addition, as an oral therapy, ibrutinib avoids the need for infusions and the potential for infusion-related reactions as it can be safely and effectively administered at home. Compared to CTP, the AE profile of ibrutinib also allows it to be safely used in elderly patients and in those with comorbidities that could otherwise limit treatment choice and tolerance [1,16,18–23]. The analysis demonstrates that ibrutinib vs. CTP is a cost-effective therapy in R/R WM patient management and the strength of this positive result was confirmed by the sensitivity analyses which show that in the majority of the simulations the ICERs fall within the WTP threshold of €60,000/LYG.

Ibrutinib is an orphan drug, the use of which contributes towards a significant improvement in the management of patients with WM. This study has also demonstrated that the drug has a positive cost-effectiveness profile in Italy.
